# Effects of a mouthwash containing *Lespedeza cuneata* extract on risk of dental caries: a randomized, placebo-controlled clinical trial

**DOI:** 10.1038/s41598-022-25162-w

**Published:** 2022-12-01

**Authors:** Yu-Rin Kim, Seoul-Hee Nam

**Affiliations:** 1 Department of Dental Hygiene, Silla University, Busan, South Korea; 2 Department of Dental Hygiene, College of Health Sciences, Kangwon National University, 346 Hwangjo-gil, Dogye-up, Samcheok-si, Gangwon-do 25945 South Korea

**Keywords:** Plant sciences, Health care

## Abstract

This study aims to evaluate the anti-caries effect of a mouthwash containing *Lespedeza cuneata* extract by confirming its effect on acid-producing capacity and bacteria causing dental caries in the oral cavity. For the same oral environments of 95 subjects who agreed to participate in this study, scaling was performed one week before the experiment. The final number of subjects included in the analysis was 82, excluding those who dropped out during the study period. A randomized placebo-controlled trial was conducted by dividing the subjects into the *Lespedeza cuneata* extract gargle group (n = 42) and the saline gargle group (n = 40). Participants in each group gargled once every day before going to bed for 5 days, and data were collected by measuring 3 times: before gargling (Baseline), immediately after gargling (Treatment), and 5 days after gargling (After 5 Days). Two trained dental hygienists confirmed the dental caries activity through the Cariview test under the guidance of a dentist. Microbiological analysis was performed to evaluate the changes in bacteria causing dental caries. By confirming the anti-caries effect in the oral environment according to the application of *Lespedeza cuneata* extract gargle, dental caries activity was found to be significantly lower from Treatment to After 5 Days (*p* < 0.05). Dental caries-causing bacteria in the upper and lower jaws were also significantly reduced (*p* < 0.05). These results confirm that *Lespedeza cuneata* extract is a natural substance with an anti-caries effect. Gargling with a mouthwash containing *Lespedeza cuneata* extract is useful in preventing dental caries and inhibiting its progression. The same mouthwash can also be used as an effective formulation for maintaining and promoting oral health.

## Introduction

Various microorganisms proliferate in the oral cavity, and dental caries and periodontal disease are among the representative oral diseases caused by these microorganisms. Dental caries is an infectious disease caused by microorganisms at all ages, and among oral bacteria, especially *Streptococcus mutans* (*S. mutans*) and *Streptococcus sobrinus* (*S. sobrinus*) are known to be major caries-causing bacteria ^1^. These bacteria produce sugar transfer enzymes such as glucosyltransferase and fructosyltransferase to synthesize insoluble glucan from saccharides and attach it to the tooth surface. The glucan is easily attached to the teeth by anaerobic bacteria to produce organic acid, which causes demineralization of minerals and causes dental caries^2^.

Effective methods of inhibiting dental caries include brushing and use of dental floss and interdental brush, toothpaste, and mouthwash^3^. In particular, mouthwash is known to be effective not only in preventing or managing oral diseases, but also in removing bad breath and in whitening teeth, so consumer interest in mouthwashes is gradually increasing^4^. Mouthwashes having various compositions including antibacterial activity for bacterial inhibition have been developed recently^5^. For this reason, mouthwashes must have excellent antibacterial activity and not only prevent the deposition of dental plaque, but also have an inhibitory effect on inflammatory substances in the oral cavity^6^. A mouthwash should penetrate well into the oral tissues, should not affect the composition of normal oral bacteria, and should maintain an effective concentration in the oral cavity for as long as possible^7^. However, commercially available mouthwashes with chemical components may develop resistant strains against oral bacteria when used for a long period of time. In the case of chlorhexidine, harmful side effects, such as coloration, dysgeusia, and inflammation of the mucous membrane, have been reported^8^. Consequently, studies are being actively conducted on antibacterial agents derived from natural products that are safe to use and can overcome the side effects caused by these chemical substances^9^.

Natural substances reported to have antibacterial activity against oral bacteria include bamboo, charcoal, lotus leaves, and jaborandi leaves^10–15^. Shiitake mushroom extract was also reported to be effective in suppressing dental caries-causing bacteria, *S. mutans* and *Actinomyces viscosus* (*A. viscosus*)^16^. Recently, dental clinical research is being conducted to evaluate the effect of using mouthwash containing natural substances on preventing dental caries. It was found that a mouthwash containing *Glycyrrhiza uralensis* extract can prevent dental caries and also lower the risk of dental caries^17^. In addition, a study by Kim and Nam reported that a natural mouthwash containing *Sambucus williamsii* var. *coreana* extract has excellent antibacterial activity against caries-causing bacteria in the oral cavity^18^. As such, clinical efficacy is being verified using natural antibiotics instead of chemical mouthwashes.

*Lespedeza cuneata* is a medicinal herb believed to increase stamina. It is called by various names depending on the region: ‘Cheonligwang’ as it is said to ‘shine even from a thousand miles away’ when one ate this herb; and ‘Daeryeokwang’ as it is said to produce ‘great power’ ^19^. Since ancient times, it has been used as an effective drug in the treatment of sexual impotence, ganacratia, cough, and asthma based on folklore. It is a perennial plant of the legume family and is distributed in Korea, Japan, China, and Taiwan. To date, *Lespedeza cuneata* has been studied for its antibacterial, anti-aging and antioxidant effects ^20^, wound healing effect^21^, skin photoaging improvement effect by UV exposure^22^, and potential as a skin whitening material^23^. Although *Lespedeza cuneata* contains various physiologically active substances along with various pharmacological actions, some studies have been conducted only on its antibacterial, antioxidant, and skin whitening effects. Studies proving the herb’s specific clinical efficacy remain insufficient. Moreover, although the antibacterial effect of *Lespedeza cuneata* extract against oral disease-causing bacteria and the stability of not having harmful effects on cells have been proven^24^, studies that observed the clinical effect of using *Lespedeza cuneata* extract on oral environment improvement have not been done.

This study aims to demonstrate the clinical antibacterial effect of a mouthwash containing *Lespedeza cuneata* extract by evaluating the dental caries risk caused by acid production and by confirming changes in major caries-causing bacteria. The functional role of the mouthwash with anti-caries effect as a natural material is also evaluated.

## Materials and methods

### Study design and protocol

**Figure 1:**
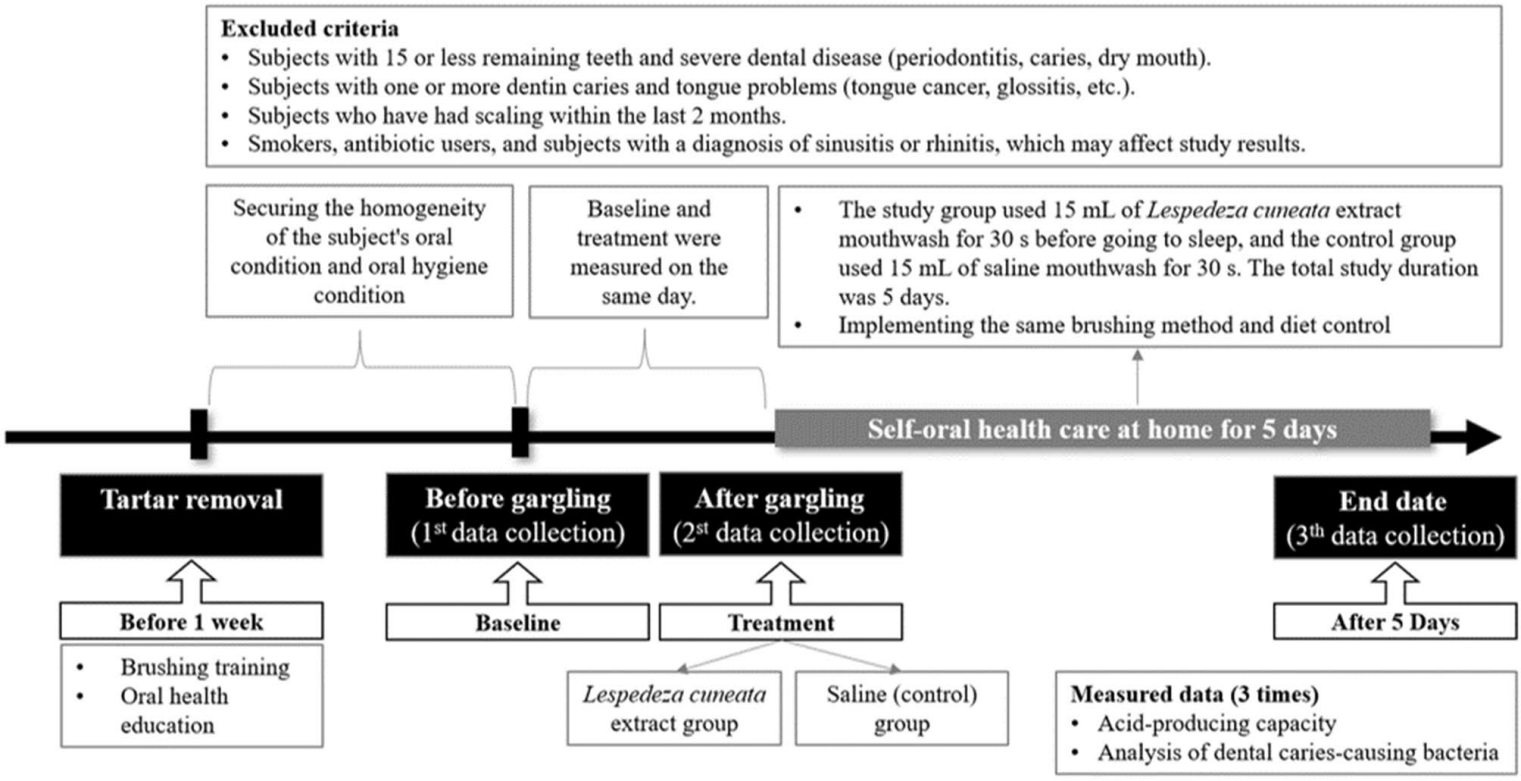
Flow chart of data measurement.

This study was a randomized placebo-controlled trial conducted at M dental clinic in Busan from October 2020 to June 2021. Two dental hygienists with 10 years of experience and who received separate training to increase raters’ reliability performed the procedure under the guidance of a dentist. For data collection, the same toothpaste and toothbrush were provided on the day the subject first came to the hospital, and they were instructed to use it for 5 days. Subjects were not allowed to know which group they belonged to, and the provided mouthwash was also labeled so that they would not know which mouthwash they were using. Oral health education was conducted for practice at home, and after 5 days, the subjects visited the dentist for a clinical examination, without doing oral hygiene behavior including brushing and gargling. In order to secure the homogeneity of the oral conditions of the subjects, scaling of all teeth was performed after oral examination by a dentist. The experiment was conducted after 1 week to restore the periodontal condition. The first day was set as the Baseline, immediately after gargling according to each group was set as Treatment, and the data measured 5 days after application was labeled as After 5 Days. For each mouthwash, 15 mL of saline mouthwash was provided to the saline gargle group while 15 mL of *Lespedeza cuneata* mouthwash was provided to the *Lespedeza cuneata* extract gargle group. All data were collected using the Cariview test and analysis of dental caries-causing bacteria, which are clinical indicators to confirm the dental caries suppression and prevention effects. Measurements for data collected were conducted in the maxillary right first molar (#16), maxillary left central incisor (#21), mandibular left first molar (#36), and mandibular right central incisor (#41) (Fig. 1).

### Inclusion and exclusion criteria

In order to secure homogeneity of the oral conditions of the subjects, those with 16 or more remaining teeth in the oral cavity and without serious dental disease (periodontal disease, dental caries, dry mouth) were selected. Additionally, those with 1 or more dentin caries and those with tongue problems (tongue cancer, glossitis, etc.) were excluded. We tried to secure reliability of the results by excluding those who had undergone scaling within the last 2 months. To ensure homogeneity of the subjects, smokers, those taking antibiotics, and those diagnosed with sinusitis or rhinitis, which could affect the study results, were excluded (Fig. 1).

### Research participants

**Figure 2:**
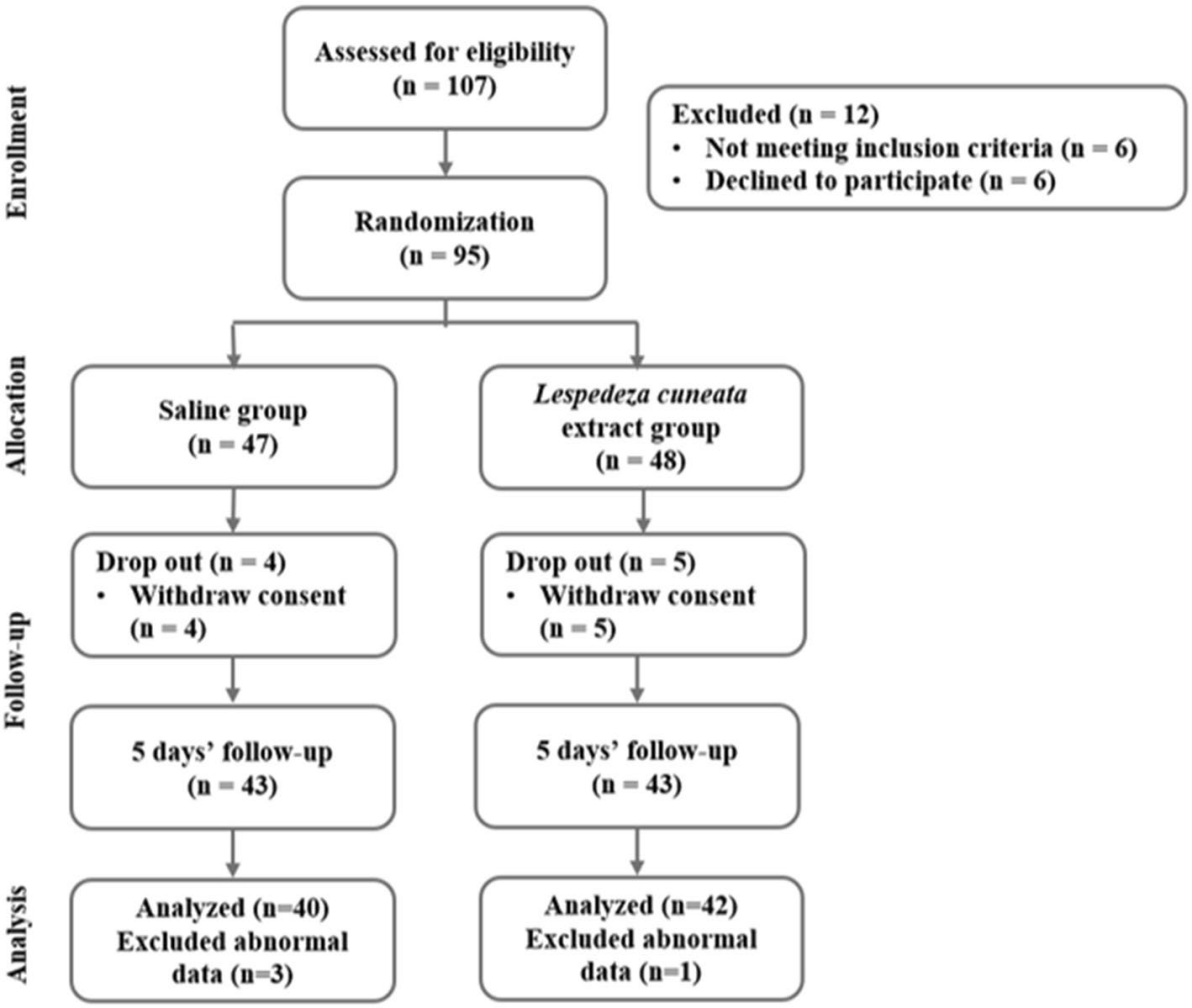
Flow chart of the study.

To determine the sample size of the study subjects, the G*Power 3.1 program was used, and the independent *t* test, which is a comparison of the two groups, was determined as the method of analysis for consistency. When the significance level was set to 0.05 by a two-sided test, power = 0.8, and effect size = 0.7, the sample size required was 68. Considering the dropout rate, we recruited 107 subjects and analyzed the final 82 subjects who remained for a total of 5 days (Fig. 2)^25^.

### Extraction of* Lespedeza cuneata*

Dried *Lespedeza cuneata* with an expiration date of less than 2 years was purchased from Cheongmyeong Co., Ltd. (Goesan, Chungcheongbuk-do, South Korea). After adding 70% ethanol to the crushed *Lespedeza cuneata*, an extract was obtained at 60 °C for 12 h. *Lespedeza cuneata* extract was filtered using qualitative filter paper, and the extract was concentrated using a rotary vacuum evaporator (N-1300E.V.S. EYELA, Rikakikai Co., Ltd., Tokyo, Japan). The *Lespedeza cuneata* was lyophilized using a freeze dryer at − 80 °C (Ilshin Lab Co., Yangju-si, South Korea). The sample was prepared in powder and used as a mouthwash containing 10 mg/mL *Lespedeza cuneata* extract.

### Acid-producing capacity

Cariview ™ kit (AIOBIO, Seoul, South Korea) is a new caries activity test kit that evaluates the acidity of organic acids secreted by microorganisms in plaque through color. Cariview ™ was used as a tool to reflect dental caries activity according to the manufacturer’s instructions ^26^. The buccal surfaces of #16 and #36 teeth in the oral cavity were rubbed with a sterile cotton swab, and then immediately put into the culture media. The medium provided by the manufacturer was 200 g Sucrose (Duksan, Kyungkido, South Korea), 20 g Tryptose (Difco, Detroit, MI, USA), 0.5 g sodium chloride (NaCl, Junsei, Kyoto, Japan), 0.2 g sodium azide (NaN3, Sigma-Aldrich, St. Louis, MO, USA.) and deionized with water to bring the total volume to 2 mL. After sufficient dissolution, the medium of the sterilized product was used. After culturing for 48 h in an incubator at 37 ℃ (10% CO_2_), 10 drops of the indicator were dropped to confirm the color change. Images taken through an optical analyzer (Allinone Bio, Seoul, South Korea) were uploaded to the manufacturer’s website and scored according to the manufacturer’s criteria. According to the criteria, a Cariview score of 0.0–40.0 indicates a low risk of dental caries activity, a score of 41.0–70.0 indicates a medium risk, and a score of 71.0–100.0 indicates a high risk of dental caries activity. The lower the Cariview score is, the lower the dental caries activity gets.

### Analysis of dental caries-causing bacteria

To obtain subgingival microbiome samples from periodontal pockets, sterile #15 paper points were placed in four sites of two maxillary teeth (anterior and posterior) and two mandibular teeth (anterior and posterior) of the subjects for 10 s and then were immediately stored at − 20 °C until just before the analysis. DNA was extracted from the collected #15 paper points using the AccuPrep Universal RNA Extraction Kit (Bioneer, Daejeon, South Korea). The extraction was performed according to the manufacturer’s instructions. OligoMix (YD Global Life Science Co., Ltd., Seongnam, South Korea) and three oligonucleotides (Table 1) that react specifically to each bacterium were used ^27^. To prepare the polymerase chain reaction (PCR) sample, 9 μL of OligoMix, 10 μL of 2 × probe qPCR mix (Takara Bio Inc., Shiga, Japan), and 1 μL of template DNA were combined. A 96-well plate with the PCR reaction sample was placed in the CFX96 Touch Real- Time PCR Detection System (Bio-Rad, Hercules, USA) to amplify the DNA. PCR conditions were as follows: initial denaturation at 95 °C for 30 s, denaturation at 95 °C for 10 s, and annealing for 30 s at 62 °C with 40 repeated cycles. The cycle threshold (Ct) value was calculated using the Bio-Rad CFX Manager Software program, and the Ct values were plotted on a standard curve for each bacterium to derive the number of copies.

**Table 1:** Primers and probes used in the real-time PCR assays.

Bacteria	Target genes	Primers/probe sets	Amplicon size (bp)
*Streptococcus mutans*	Mannitol-specific enzyme II (mtlA) gene	5′-CAGCGCATTCAACACAAGCA-3′ 103 5′-TGTCCCATCGTTGCTGAACC-3′ 5′-HEX-TGCGGTCGTTTTTGCTCATGG-BHQ1–3′	103
*Streptococcus sobrinus*	16S ribosomal RNA gene	5′-GTACAACGAGTCGCAAGCCG-3′ 149 5′-TACAAGGCCCGGGAACGTAT-3′ 5′-FAM-TAATCGCGGATCAGCACGCC-BHQ1–3′	149
*Actinomyces viscosus*	Sialidase (nanH) gene	5′-GCTCCCTCATGCTCAACTCG-3′ 5′-GATGATCTGGGCGTTGTCCA-3′ 5′-Texas Red-GAGCCGGTCCCCGACAAGAA-BHQ2–3′	140

### Statistical analysis

The data were analyzed with SPSS ver. 24.0 (IBM SPSS Statistics 24.0, SPSS Inc., Chicago, IL, USA). Independent *t* test, chi-square test, and Fisher’s exact test were performed to confirm the difference in demographic characteristics between the two groups. In addition, the temporal change in the dental caries activity test according to the application of gargles in the two groups was analyzed with independent *t* test and one-way ANOVA, followed by the Tukey test as a post-hoc test. Fisher’s exact test was performed since there was a value less than 5 to compare the means at Baseline and After 5 Days by group for the risk of dental caries activity. Finally, the mean comparison of dental caries-related bacteria at the maxilla and mandible in the temporal flow between the two groups was performed with independent *t* test and one-way ANOVA, followed by the Tukey test as a post-hoc test. The significance level was *p* = 0.05 for the significance test between groups.

### Ethics approval and consent to participate

This study was approved for human studies (KWNUIRB-2020-07-007–002, Chuncheon, South Korea) and registered as a clinical trial on the WHO International Clinical Trial Registry Platform (ICTRP) (13/06/2022, registration number: KCT0007379; https://cris.nih.go.kr/cris/search/detailSearch.do/22017). The purpose and procedures of this study were explained to all participants, who were informed that they would not be penalized for refusing to participate and that they could withdraw from the study at any time. Additionally, this study was conducted in accordance with the International Council for Harmonization of Technical Requirements for Pharmaceuticals for human use (ICH) guideline.

## Results

### Study population

The initial number of subjects in this study was 107, but 12 were excluded for not meeting the requirements of the study or for refusing to participate, bringing the final number of 95 subjects. We randomly divided the subjects into the saline gargle group (47 subjects) and the *Lespedeza cuneata* extract gargle group (48 subjects). As the study progressed for 5 days, there were 4 dropouts in the saline gargle group and 5 dropouts in the *Lespedeza cuneata* extract gargle group. After 5 days, the data of 43 subjects were collected from two groups. Excluding subjects with outliers among the collected data, data from 40 subjects in the saline gargle group and 42 subjects in the *Lespedeza cuneata* extract gargle group were analyzed (Fig. 2). There were more women than men in both groups, and the average age was 27.15 years in the saline gargle group and 27.86 years in the *Lespedeza cuneata* extract gargle group. There were more subjects without systemic disease and more subjects who were single in both groups. There was no significant difference in demographic variables between the two groups (Table 2).

**Table 2:** Characteristics of the subjects in the two groups (n = 82).

Characteristics	N (%)		
	Saline (n = 40)	*Lespedeza cuneata* extract (n = 42)	*p* value
*Gender			
Male	6 (15.0)	8 (19.0)	0.626
Female	34 (85.0)	34 (81.0)	
^￥^**Age (mean ± SD)**	27.15 ± 7.97	27.86 ± 8.32	0.696
*Systemic disease			
No disease	37 (92.5)	38 (90.5)	1.000
Have a disease	3 (7.5)	4 (9.5)	
*Marriage			
Single	34 (85.0)	34 (81.0)	0.626
Married	6 (15.0)	8 (19.0)	

^￥^*p* values are determined by independent *t* test, *p values are determined by chi-square test & Fisher’s exact test (*p* < 0.05). Values are means ± standard deviations.

### Evaluation of dental caries risk

Dental caries activity was quantified and measured based on the pH value caused by the acid production of oral bacteria, and the dental caries risk was evaluated based on the Cariview score. Results showed no significant difference in Baseline between the two groups (*p* > 0.05). Cariview scores and risk were lower in the *Lespedeza cuneata* extract gargle group at Treatment and After 5 Days, and these results were significantly different from the saline gargle group (*p* < 0.05). There was also no significant difference in the temporal change according to the application of the two gargles in the saline gargle group, but the *Lespedeza cuneata* extract gargle group showed a significant decrease with time at Treatment and After 5 Days compared to the Baseline (Table 3). Based on the risk distribution analysis, the difference in the distribution was not significant during the application period (*p* > 0.05) in the saline gargle group. In the *Lespedeza cuneata* extract gargle group, the low risk and moderate risk increased 5 days after application, and the high risk was not observed. (*p* < 0.05) (Table 4).

**Table 3:** Changes in the Cariview scores of the two groups.

Variables	Group	Mean ± SD			*p value
		Baseline	Treatment	After 5 Days	
Cariview scores	Saline	59.66 ± 5.72	52.50 ± 7.34	50.59 ± 6.62	0.058
	*Lespedeza cuneata* extract	59.71 ± 7.32	41.43 ± 4.91	41.49 ± 3.51	**0.000**
	^￥^*p* value	0.989	**0.001**	**0.003**	
Risk	Saline	2.23 ± 0.21	2.20 ± 0.20	2.14 ± 0.18	0.711
	*Lespedeza cuneata* extract	2.30 ± 0.23	1.90 ± 0.15	1.78 ± 0.21	**0.000**
	^￥^*p* value	0.568	**0.002**	**0.001**	

^￥^*p* values are determined by independent *t* test, *p values are determined by one-way ANOVA and Tukey tests (*p* < 0.05).; Values are means ± standard deviations.; significant (bold); 0.0–40.0, low risk; 41.0–70.0, medium risk; 71.0–100.0, high risk; 1, low-risk; 2, medium-risk; and 3, high-risk.

**Table 4:** Distribution of the subjects according to their risk differences (n = 82).

Group	Risk	N (%)		*p value
		Baseline	After 5 Days	
Saline	Low	0 (00.0)	0 (00.0)	0.425
	Moderate	32 (80.0)	33 (82.5)	
	High	8 (20.0)	7 (17.5)	
*Lespedeza cuneata* extract	Low	0 (00.0)	4 (9.5)	**0.004**
	Moderate	30 (71.4)	38 (90.5)	
	High	12 (28.6)	0 (00.0)	

*p values are determined by Fisher’s exact test (*p* < 0.05); significant (bold).

### Activity of dental caries-causing bacteria

Bacteria that cause dental caries, namely *S. mutans*, *S. sobrinus*, and *A. viscosus,* were detected. When comparing the saline gargle group and the *Lespedeza cuneata* extract gargle group for *S. mutans*, there was a significant decrease at Treatment and After 5 Days in the maxilla. There was a significant difference at Treatment and After 5 Days compared to Baseline (*p* < 0.05) in the mandibular, having no *S. mutans* observed at Treatment and After 5 Days. *S. sobrinus* decreased showing a significant difference between the two groups in the maxilla and mandibular at Treatment and After 5 Days (*p* < 0.05). *A. viscosus* showed a significant difference between the two groups only in the mandibular at Treatment and After 5 Days (*p* < 0.05).

In the case of the saline gargle group, the only change with time from Baseline to Treatment and After 5 Days was a significant decrease in *A. viscosus* in the mandibular (*p* < 0.05). In the case of the *Lespedeza cuneata* extract gargle group, all three bacteria showed a significant decrease in the maxilla and mandibular (*p* < 0.05) (Table 5).

**Table 5:** Clinical outcomes observed between the groups.

Variables		Group	Mean ± SD			
			Baseline	Treatment	After 5 Days	*p value
*Streptococcus mutans*	Maxilla	Saline	1593.41 ± 1595.99^a^	1528.42 ± 1159.33^a^	1645.71 ± 991.88^a^	0.992
		*Lespedeza cuneata* extract	1379.38 ± 885.53^a^	387.00 ± 586.87^b^	133.24 ± 160.79^b^	**0.001**
		^￥^*p *value	0.763	**0.035**	**0.014**	
	Mandibular	Saline	51.96 ± 25.05^a^	23.00 ± 21.23^a^	30.26.23.30^a^	0.087
		*Lespedeza cuneata* extract	351.52 ± 26.59^a^	0.00 ± 0.00^b^	0.00 ± 0.00^b^	**0.000**
		^￥^*p *value	**0.006**	**0.012**	**0.011**	
*Streptococcus sobrinus*	Maxilla	Saline	1285.59 ± 565.02^a^	525,664.93 ± 269,454.10^a^	333,916.70 ± 173,500.74^a^	0.238
		*Lespedeza cuneata* extract	441,074.82 ± 227,322.03^a^	104,063.38 ± 60,096.53^b^	96,797.42 ± 53,335.40^b^	**0.000**
		^￥^*p *value	0.370	**0.000**	**0.004**	
	Mandibular	Saline	412,635.35 ± 269,375.13^a^	351,940.78 ± 258,647.71^a^	369,800.30 ± 105,396.48^a^	0.882
		*Lespedeza cuneata* extract	385,448.22 ± 185,448.29^a^	120,766.60 ± 80,220.88^b^	92,382.15 ± 50,117.54^b^	**0.000**
		^￥^*p *value	0.834	**0.033**	**0.000**	
*Actinomyces viscosus*	Maxilla	Saline	1,105,848.32 ± 1,366,783.90^a^	593,704.89 ± 182,469.48^a^	554,354.58 ± 165,586.36^a^	0.382
		*Lespedeza cuneata* extract	1,239,751.44 ± 513,808.10^a^	487,415.71 ± 138,920.57^b^	653,762.11 ± 363,020.09^b^	**0.002**
		^￥^*p *value	0.812	0.228	0.593	
	Mandibular	Saline	686,684.63 ± 150,353.47^a^	436,532.76 ± 132,172.27^b^	427,029.95 ± 15344061^b^	**0.016**
		*Lespedeza cuneata* extract	874,194.88 ± 323,708.09^a^	266,444.79 ± 86,192.13^b^	192,749.61 ± 63,459.80^b^	**0.000**
		^￥^*p *value	0.287	**0.013**	**0.004**	

^￥^*p* values are determined by independent *t* test, *p values are determined by one-way ANOVA and Tukey tests (*p* < 0.05).; Values are means ± standard deviations; significant (bold).

## Discussion

Dental caries is a chronic disease caused by the start of demineralization by acid generated in the dental plaque and is a common disease that 80–90% of Koreans suffer from^28^. Prevention of dental caries before they occur is important, and for this purpose, dental plaque management is critical. The common and representative method of dental plaque management is brushing to remove the attached dental plaque through physical friction. The use of mouthwash, as another dental plaque management method, is in liquid form and has antibacterial or tooth surface remineralization effect by the function of chemical agents. This makes mouthwash useful in oral care for infants, orthodontic patients, or patients with mobility problems^29,30^. However, since products containing chemical agents have been reported to be toxic or cause tooth discoloration^31^, research on alternative products continues. In traditional medicine, natural substances have been proposed as viable alternatives for synthetic chemicals ^32^. Nam ^24^ evaluated the effect of *Lespedeza cuneata* extract on human keratinocytes (HaCaT) that normally inhabit the epithelial mucosa in the oral cavity. After 3 h of application of *Lespedeza cuneata* extract, the cell viability was 51% at a concentration of 10 mg/mL. *Lespedeza cuneata* extract at 10 mg/ml has a half maximal inhibitory concentration (IC50), so it can act as a safe and excellent natural antibiotic.

Therefore, this randomized placebo-controlled trial confirmed the changes in dental caries risk and amount of dental caries-related bacteria when a mouthwash containing *Lespedeza cuneata* extract, a natural substance with proven antibacterial effects and biostability, is used as gargle. Cariview scores were lowered when *Lespedeza cuneata* extract gargle was applied, and the dental caries risk was also significantly reduced. This means that the lower the Cariview score, the higher the pH value becomes, indicating that acid production did not occur and further resulting in reduced dental caries risk. Additionally, in the case of saline gargle, there was still a high risk after 5 days of gargling, but in the case of the mouthwash containing *Lespedeza cuneata* extract, high risk was not observed, while the low risk and moderate risk increased. As an effective anti-caries mouthwash, it can lower the risk of dental caries just by gargling for 5 days.

The mouthwash also showed excellent effect of inhibiting bacteria that cause dental caries. Compared to dental clinical studies on *Glycyrrhiza uralensis* extract ^17^ and *Sambucus williamsii* var. *coreana* extract ^18^ for *S. mutans,* which are the most common cause of dental caries, *S. mutans* was not detected at all in the mandibular from immediately after gargle of *Lespedeza cuneate* extract used in this study. It was also not detected even after 5 days of use since the antibacterial effect persisted. *S. sobrinus* also showed a significant and continuous decrease over time from immediately after gargling using the mouthwash containing *Lespedeza cuneata* extract to 5 days after. *S. sobrinus* is known to increase the incidence of dental caries due to its strong acid-producing capability when coexisting with *S. mutans*^33^. It is also associated with systemic diseases such as pneumonia, hemorrhagic stroke, acute endocarditis, meningitis, temporomandibular arthritis, otitis media, sepsis, and ulcerative colitis^34^. The use of *Lespedeza cuneata* extract shows an antibacterial effect against *S. sobrinus* and *S. mutans*, so it can be used for the prevention of oral diseases as well as systemic diseases. *A. viscosus* is known to be involved in oral diseases such as root caries and periodontitis as well as systemic diseases such as sepsis and endocarditis^35^. Compared to saline gargle, *A. viscosus* detected in the mandibular decreased immediately after using the mouthwash containing *Lespedeza cuneata* extract and after 5 days of use. In the case of the maxilla, there was no significant difference although bacteria tend to decrease at Treatment and After 5 Days. There was immediate effect in the mandibular because of retention of mouthwash, whereas the maxilla had less contact than the mandibular, resulting in insufficient antibacterial effect. Gargling with *Lespedeza cuneata* extract for 5 days showed an excellent antibacterial action in the oral cavity with a distinct reduction in the three caries-causing bacteria detected, confirming that use of the mouthwash can prevent dental caries.

Based on the above results, the use of mouthwash containing *Lespedeza cuneata* extract significantly reduces the risk of dental caries activity by inhibiting acid production. It also acts as a natural antibiotic with anti-dental caries function by inhibiting and killing dental caries-causing bacteria. Therefore, a mouthwash containing *Lespedeza cuneata* extract can be effectively used by all age groups as a self-care method for dental caries prevention. It is a safe natural substance, and it can be used in various ways to prevent caries. This study, however, has the following limitations. Since the mouthwash presented in this study was not compared with commercially available mouthwashes, a comparative study needs to be conducted in the future. The period of this study is also relatively short, so we intend to confirm the changes according to long-term use in the future. Furthermore, we plan to conduct research on the development of a mouthwash with optimal formulation by mixing various natural extracts and confirming their synergistic effects with natural mixtures.

## Conclusion

Since a mouthwash containing *Lespedeza cuneata* extract reduces the risk factor of caries activity by inhibiting acid production in the oral cavity, it is expected to have high potential for use as a mouthwash containing natural material with excellent anti-carious properties. In addition, the excellent antibacterial action against dental caries-causing bacteria also addresses the side effects of using mouthwash containing conventional chemical components. Therefore, further research on the development of various oral prevention products with antibacterial effects and preventing the risk factor of dental caries using *Lespedeza cuneata* extract will contribute to the improvement of oral health care effect and ultimately promote public oral health.

## Data Availability

The data sets generated and/or analyzed during the current study are not publicly available for reasons of personal and organizational integrity. However, these are available from the corresponding author upon reasonable request.

## Abbreviations

*S. mutans*: *Streptococcus mutans*
*S. sobrinus*: *Streptococcus sobrinus*
*E. nodatum*: *Eubacterium nodatum*
*A. viscosus*: *Actinomyces viscosus*
